# New high‐throughput fully automated BD‐Tau immunoassay

**DOI:** 10.1002/alz70856_101595

**Published:** 2025-12-25

**Authors:** Miklos Szabo, Ben Schlichtmann, Jeff Todtleben, Kara Curtis, Laura Mediger, Marnie Wallin, Mikaela Nichkova‐Doseva

**Affiliations:** ^1^ Beckman Coulter Inc., Chaska, MN, USA; ^2^ Beckman Coulter Inc, Chaska, MN, USA

## Abstract

**Background:**

The analytical performance characteristics of the BD‐Tau (brain‐derived Tau) immunoassay currently under development on the Beckman Coulter Access 2 and DxI 9000 Access analyzers is described. BD‐Tau measurement is being under investigation as a tool to differentiate between multiple types of dementia and as an indicator of neurologic injury.

**Method:**

The prototype BD‐Tau assay is a two‐step sandwich assay utilizing an anti‐N‐terminal Tau monoclonal (mAb) antibody/alkaline phosphatase conjugate and paramagnetic particles coated with an anti‐BD‐Tau mAb. The assay time to first result is ∼35 minutes. Sixty‐nine EDTA plasma and serum samples from subjects with Alzheimer's disease (AD; *n* = 29) or frontotemporal dementia (FTD; *n* = 29) and 20 age‐matched healthy, normal control subjects were evaluated. A method comparison was performed between Access 2 and DxI 9000.

**Result:**

The prototype BD‐Tau assay has a targeted analytical measuring range of up to approximately s2000 pg/mL. 100% of normal control samples were quantified, with low dose variability (<4.0% coefficient of variation). Comparison of the prototype BD‐Tau assay on DxI 9000 and Access 2 yielded a Passing‐Bablok slope of 1.16 (r=0.999) and an intercept of ‐0.05 pg/mL. The dose ratios of the median of AD over the median of normal samples for Access 2 and DxI 9000 were 2.0 and 1.6, respectively; AD patient median dose values were 5.6 pg/mL and 4.5 pg/mL, respectively. Normal sample median dose value was 2.8 pg/mL on both platforms. On Access 2 and DxI 9000, the FTD median dose values were 6.2 pg/mL and 5.4 pg/mL, respectively; the dose ratios of median FTD over median of normal samples were 2.2 and 1.9, respectively. This assay can be used for both plasma and serum. Comparison of serum against EDTA plasma on the DxI 9000 analyzer yielded a Passing‐Bablok slope of 0.96 (r=0.988) and an intercept of 0.02 pg/mL across 47 matched samples, including AD, FTD, and controls.

**Conclusion:**

The first high‐throughput, fully automated BD‐Tau assay provides fast, highly sensitive, precise results on the Beckman Coulter Access 2 and DxI 9000 platforms. It presents high selectivity between individuals without neurodegenerative disease versus AD and FTD subjects.

